# Comparison of Eye and Face Features on Drowsiness Analysis

**DOI:** 10.3390/s22176529

**Published:** 2022-08-30

**Authors:** I-Hsi Kao, Ching-Yao Chan

**Affiliations:** California Partners for Advanced Transportation Technology, University of California, Berkeley, CA 94804, USA

**Keywords:** deep learning, drowsiness analysis, Grad-CAM, KNN-Sigma

## Abstract

Drowsiness is one of the leading causes of traffic accidents. For those who operate large machinery or motor vehicles, incidents due to lack of sleep can cause property damage and sometimes lead to grave consequences of injuries and fatality. This study aims to design learning models to recognize drowsiness through human facial features. In addition, this work analyzes the attentions of individual neurons in the learning model to understand how neural networks interpret drowsiness. For this analysis, gradient-weighted class activation mapping (Grad-CAM) is implemented in the neural networks to display the attention of neurons. The eye and face images are processed separately to the model for the training process. The results initially show that better results can be obtained by delivering eye images alone. The effect of Grad-CAM is also more reasonable using eye images alone. Furthermore, this work proposed a feature analysis method, K-nearest neighbors Sigma (KNN-Sigma), to estimate the homogeneous concentration and heterogeneous separation of the extracted features. In the end, we found that the fusion of face and eye signals gave the best results for recognition accuracy and KNN-sigma. The area under the curve (AUC) of using face, eye, and fusion images are 0.814, 0.897, and 0.935, respectively.

## 1. Introduction

A substantial proportion of car accidents can be attributed to human negligence or errors. Operating a motor vehicle while fatigued or sleepy is commonly referred to as drowsy driving [1]. Drowsiness makes drivers less attentive, slows reaction time, and affects a driver’s ability to make decisions [2]. A driver might not even know when he or she is fatigued because signs of fatigue are hard to identify. Some people may also experience micro-sleep—short, involuntary periods of inattention. In the 4 or 5 s a driver experiences micro-sleep, at highway speed, the vehicle will travel the length of a football field [3].

Drowsy driving is a serious traffic problem in the United States [4]. National Highway Traffic Safety Administration (NHTSA) reported that 697 fatalities in motor vehicle crash involved drowsy drivers in 2019 [5]. Drowsy driving fatalities were 1.9 percent of total driving fatalities in 2019. A study by American Automobile Association Foundation for Traffic Safety found that observable driver drowsiness was present in an estimated 8.8 percent to 9.5 percent of all crashes [6]. These proportions are significant, several times higher than statistics published by NHTSA. The Governors Highway Safety Association issued a report concluding that the estimated annual societal cost of fatigue-related fatal and injury crashes was $109 billion [7]. This figure does not include property damage. It can be reasoned that if human drowsiness detection and alert systems were provided on all powered vehicles, there could be a significant reduction in human casualties and repair of damaged resources each year.

Many publications discuss building and designing drowsiness recognition systems [8,9,10]. Methodologies for driver drowsiness analysis can be divided into three categories: (a) behavioral parameter-based techniques [11,12,13], (b) vehicular parameter-based techniques [14,15,16], and (c) physiological parameter-based techniques [17,18,19]. The electroencephalogram (EGG) analysis can fairly accurately recognize the drowsiness of participants [20,21]. However, measuring the EGG from the drivers is unsuitable for implementation in motor vehicles. Most of the previous studies in this domain fall in the first category, based on behavioral parameters, which is a way to detect drowsiness based on a non-invasive method. Human drowsiness is indicated by behavior parameters such as blinking, head position, facial expression, yawning, and eye-closing rate.

In [22], a survey of drowsiness technology is presented. The author classified the technology of the methods into behavioral-based [23,24,25,26,27,28,29,30,31,32,33], physiological-based [34,35,36], vehicular-based, and hybrid [36,37]. Several methods are included in the behavioral-based technologies, such as eye status recognition (open or close), blinking frequency, yawning, and head motion. The signal source of physiological-based methods includes, but is not subject to, EGG signals, Electrocardiogram signals, Photoplethysmogram, Heart Rate Variability, Electrooculogram signals, and electromyogram signals. In vehicular-based technologies, researchers used to detect driver drowsiness by using sensors placed on the various parts of the vehicle, such as the steering wheel and the acceleration pedal. The common two vehicular measures used are the steering wheel movement and the standard deviation of lane position. Hybrid techniques are a combination of several techniques, such as a combination of behavioral and physiological techniques or a combination of behavioral and vehicular-based techniques.

In [38], an ensemble of four models includes AlexNet [39], FaceNet [40], FlowImageNet, and ResNet [41] is presented. The use of AlexNet is to extract the environment feature; the use of FaceNet is to extract the face feature; the use of FlowImageNet is used to extract the behavioral feature, and the use of ResNet is to extract the hand gestures. After all the predictions of the four models, the average prediction value is calculated and measures the drowsiness of the participants. The author implemented several models for the feature extraction; however, there is no extraction comparison and feature analysis in the work. In our work, not only is a useful model presented, but also the attention to the model and the feature extraction analysis is discussed.

In [42], the author presented a hand-crafted feature extraction method for extracting the drowsiness feature. The authors implemented Pyramid Multi-Level for the face representation and used a Histogram of Oriented Gradients [43], Covariance descriptor [44], and local binary pattern [45] to extract the feature of each image block from the Pyramid Multi-Level. After the feature extraction, PCA is used to decrease the feature dimension and three SVM are used to classify the extracted feature. The author produced great work in hand-crafted features and shows advantage accuracy rates against deep learning methods such as Alexnet [39], VGGFaceNet [46], and ResNet [41]. However, the author did not analyze the hand-craft feature and describe the importance of the extracted features. All of the above studies lack an explanation of the characteristics of neural networks. This study not only established an effective neural network but also found that inputting a full face would cause the neural network to be distracted. Therefore, it can also be interpreted that the hand-crafted feature method of [42] can outperform the training method of neural networks.

In [47], a real-life drowsiness dataset (UTA-RLDD) was presented for the task of multistage drowsiness detection. The cases in the dataset target not only extreme and easily visible cases but also subtle cases of drowsiness. There are sixty participants in the dataset with a frequency rate of under thirty. An end-to-end baseline method is presented with the temporal relationship between blinks for multistage drowsiness detection. This work utilizes this dataset to present the drowsiness analysis.

In [48], a compression deep learning model is implemented on JetsonTK1 for driver drowsiness detection. The model combines a pre-trained model, Multi-Task Cascaded Convolutional Networks (MTCNN) [49], and a designed model proposed by the author. MTCNN is implemented to detect the face and other detection landmarks in the image, and the designed network detects drowsiness. The detection landmarks include eyes and mouth. The implemented drowsiness detection has three outcomes: normal, yawning, and drowsy. The experimental results show that the accuracy is reduced from 93.84% to 89.46% after compression. However, the frame per second (FPS) increased from 12.5 to 14.9.

In [50], the author aims to detect the drowsiness in the UTA-RLDD by deep learning. In the first phase, Haar-cascade [51] is used to detect faces in the image. After extracting the face image, the face image is processed by a stacked convolutional neural network (CNN) [39]. After training by the stacked CNN, the model reached an acceptable accuracy on UTA-RLDD. Moreover, the system maintains the accuracy rate by using a customized dataset and learning transfer on the model.

In the above-referenced studies, some authors extracted the blink event as a neural network input since blinking events have been highly correlated with human drowsiness. However, other facial feature representations may need to be analyzed together with the blinking event to improve the drowsiness detection system. We believe the complete consideration of relevant facial features can effectively increase detection accuracy. We hope this work can find innovative definitions via a combination of facial features. The results of this research can advance the understanding of drowsiness. Ultimately, we hope it will lead to preventive measures to improve traffic safety.

In addition, some studies chose to use the entire driver’s face image for deep learning feature extraction. However, these studies did not confirm whether the neural network was correctly learning drowsiness features or face recognition. In order to demonstrate the discriminative ability of the neural network, in this work, gradient-weighted class activation mapping (Grad-CAM) is implemented to analyze the learning ability of the neural network further. Furthermore, the homogeneous concentration and heterogeneous separation of the extracted features are compared for analysis by feature visualization and K-nearest neighbors Sigma (KNN-Sigma).

This work not only uses face images for drowsiness detection but also validates drowsiness detection using only eye images. The results show greater accuracy than only using face images, and FPS can be improved using only eye images. We also fuse face images with eye images for training. The results show that the fused images can obtain the best results in recognition and KNN-Sigma. Therefore, zooming in on the features of the eyes helps the neural network recognize drowsiness features.

The contributions of this work are as follows:

1. Design deep learning model structure to detect human drowsiness based on UTA-RLDD.
2. Propose and build a model that can detect drowsiness using only eye features.
3. Implement Grad-CAMs in the deep learning models for analyzing the drowsiness feature learning ability of the models.
4. Present KNN-Sigma and implementation of feature visualization for deep learning models for analyzing the homogeneous concentration and heterogeneous separation.

The remainder of this paper is organized as follows. In Section 2, data pre-processing of the public dataset, UTA-RLDD, is presented. In Section 3, the designed model is presented. In addition, the concept of Grad-CAM, feature visualization, and KNN-Sigma are presented. In Section 4, the experimental results and the discussions are presented. Finally, Section 5 concludes the work and suggests a future research topic.

## 2. Data and Data Pre-Processing

In this section, we present the database used for the study and the steps taken to pre-process the data for our model.

### 2.1. Data

The dataset used in this work is UTA-RLDD. It was a dataset recorded by the University of Texas at Arlington. There are 60 healthy participants in the dataset. Every participant provides three types of status: alertness, low vigilance, and drowsiness. Therefore, the dataset contains 180 videos with 30 h of RBG frame. Fifty-one males and nine females, all over 18 years old, participated in this experiment. Among the 180 videos, 21 wore glasses, and 72 had considerable facial hair. The videos were developed in reality scenes with different backgrounds. Every scene was captured by the participants’ phone or webcam with an FPS under 30, which is representative of the frame rate expected of normal cameras used by the general population. Therefore, none of the videos in this dataset have a fixed FPS and resolution. We believe that such a setting can instead increase the generality of the model. Models are not limited to images that require a specific FPS or resolution to effectively detect drowsiness. The total size of the dataset is 111.3 GB.

The three types of drowsiness status are defined according to the Karolinska sleepiness scale (KKS) [52]. The KKS is a scale that can be used to self-assess fatigue, describing nine different drowsiness levels. The description is shown in Table 1. According to the KKS, the classification of the drowsiness status should be divided into nine levels, but UTA-RLDD only defines drowsiness in three levels. The alert level, low vigilance level, and drowsy level of UTA-RLDD are defined according to the description of the Level 1, 2, 3, Level 6, 7, and level 8, 9 of the KKS, respectively. This work only considered the definition of alert and drowsy levels defined in URA-RLDD.

Some participants did not wish to publish their faces in papers or publications. This work abides by the rules of the dataset and only publishes images of participants willing to be published.

### 2.2. Data Pre-Processing

**Figure 1:**
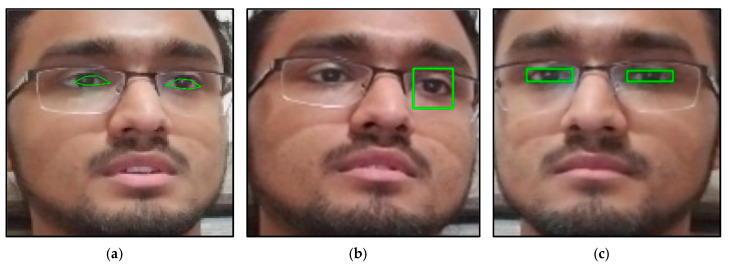
The eye detection results: (**a**) dlib facial landmark, (**b**) Haar-cascade object detection, and (**c**) MediaPipe face mesh. (The ID number of the participants is nine in the UTA-RLDD. The participant claims to allow his image to be published in research papers and publications.).

Before processing the dataset through the neural network, the images should be pre-processed to extract the face and eye images. The authors use the MTCNN and Haar-cascade to extract the face or eye images in [48,50]. However, we found that in some images, the MTCNN and Haar-cascade are unreliable in detecting the eyes. In order to choose a stable function for the detection of eyes, a comparison of three different methods, which include dlib facial landmark [53], Haar-cascade object detection, and MediaPipe face mesh [54], is shown in Figure 1. Figure 1a shows the results of the dlib facial landmark; Figure 1b shows the results of Haar-cascade object detection; Figure 1c shows the results of MediaPipe face mesh.

The dlib facial landmark is not an eye detection method but a facial landmark detection method. With this method, the eye landmark detection results show an offset while detecting participants wearing glasses, as shown in Figure 1a. The model chosen in this work for dlib was trained by Álvarez Casado [55]. The Haar-cascade object detection shows no offset in the anchor box. However, while the participants wear glasses, the detection misses one or both eyes in some frames, as shown in Figure 1b. The model of Haar-cascade chosen in this work is “haarcascade_eye_tree_eyeglasses.xml”.

The MediaPipe face mesh showed the best result in detecting eyes. Even though the participants wear glasses, the detection is stable and correct, as shown in Figure 1c. However, the MediaPipe face mesh is not an eye detection method but a face mash detection method. In order to make the function into an eye detection method, the maximum and minimum of the facial landmark of the left and right eyes are extracted to draw the anchor box. Since the MediaPipe face mesh showed the best results, it was chosen to be implemented as a pre-trained model in the pre-processing of extracting the face and eye images. The model extracted all the face and eye images from the dataset, except when the eyes were completely covered.

The method of detecting the face in MediaPipe face mesh is the Blaze Face Detector (BFD) [56]. The BFD is a lightweight model that detects faces in images. A custom encoder, Single Shot Detector (SSD) architecture [57], is implemented in the BFD that can be used in the first steps of face-related computer vision applications. The BFD is designed for front-facing cameras on mobile devices, where the captured face usually occupies a more significant portion of the frame. The BFD may have difficulty detecting faces at a distance. However, in our experimental environment, the distance between the face and the camera is not large enough to affect the accuracy of the BFD.

**Figure 2:**
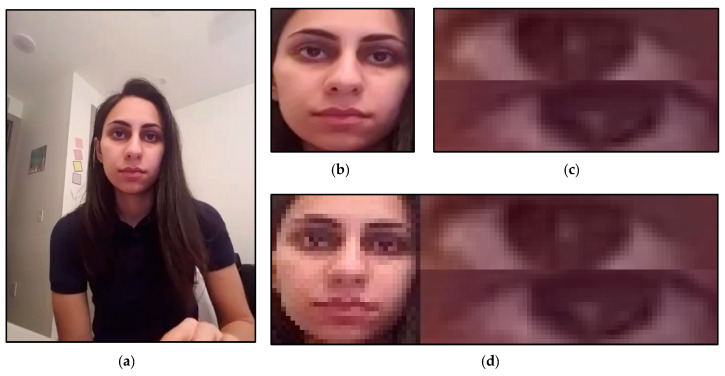
The pre-processed results for the neural network input: (**a**) origin input, (**b**) face extraction result, (**c**) eye extraction result, and (**d**) fusion result. (The ID number of the participants is seventeen in the UTA-RLDD. The participant claims to allow her image to be published in research papers and publications.).

Three models, to be explained in the next section, are designed in this study that works with different image sets with corresponding input sizes. The image sets are shown in Figure 2. Figure 2a shows the original image; Figure 2b shows the extracted face image; Figure 2c shows the extracted eye image; Figure 2d shows the fusion image. Therefore, after the face extraction, the face image will be resized to (32, 32) or (28, 28), depending on the input size of the designed model. If the face image is an independent input of the model, the face image will be resized to (28, 28), as shown in Figure 2b, and if the face image is part of the fusion image of the model, the face image will be resized to (32, 32) and stack with the eye image as shown in Figure 2d. After the eye extraction, the left and right image was resized to (16, 64) and stacked into one image, as shown in Figure 2c. If the eye image was considered a fusion image, it was further stacked with the face image, as shown in Figure 2d, and the fusion image size will be (32, 96).

## 3. Methods

**Figure 3:**
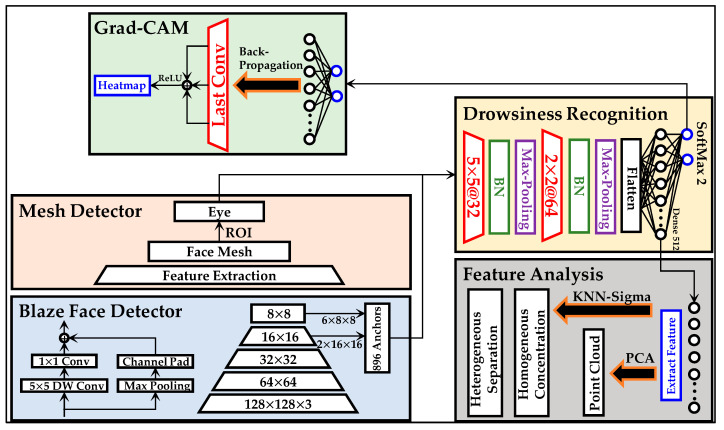
The experimental diagram in this work.

The experiments in this work are divided into four phases: pre-processing, drowsiness recognition, Grad-CAM, and KNN-Sigma. The pre-processing is discussed in the previous section. This section presents the methods of drowsiness detection, Grad-CAM, and KNN-Sigma. The diagram in Figure 3 illustrates the total framework of methodologies.

### 3.1. Drowsiness Recognition Model

The drowsiness detection method includes three models, as shown in Figure 3. The structure of the models is the same, but the input sizes of the models are different. The input contains three structure types: face, eye, and fusion image, as explained and shown in Figure 2b–d, respectively.

The model contains two CNN, two batch normalization (BN) [58], two Max-pooling [59], and two fully connected layers (FCN). The equation of the CNN is shown as follows:

(1) $$G\left(m,n\right)=f\left(f*h\right)\left[m,n\right]=\underset{j}{\sum }\underset{k}{\sum }h\left(j,k\right)f\left(m-j,n-k\right)$$

where f is the input image, h is the convolutional kernel, m is the row of the image, n is the column of the image, j is the row of the convolutional kernel, and k is the column of the convolutional kernel. The stride of the CNN is set as one. The padding method in this work is the “same,” meaning an extra edge will be added to the inputs of the convolutional layer. The size of the additional edge should fit the following function:

(2) $$p=\frac{size\left(h\right)-1}{2}$$

where p is the padding size and $size\left(h\right)$ is the size of the convolutional kernel. The activation function of CNN is LeakyReLU [60], as shown following:

(3) $$f\left(x\right)=\left\{\begin{array}{c} \alpha x,\;|x<0\\ \;\;\;x,\;|x\geq 0 \end{array}\right.$$

where *x* is the input value, and α is the slope while the input x is lower than zero. The α in this work is set as 0.3.

After every CNN, a BN is connected to avoid the vanishing gradient. The formula of BN is shown as follows:

(4) $$Y=\gamma \frac{X-\mu_{x}}{\sqrt{\sigma_{x}^{2}-\epsilon }}+\beta$$

where Y is the output of the BN, X is the input of the BN, $\mu_{x}$ is the mean of the X, $\sigma_{x}^{2}$ is the variance of X, ϵ is a minimal value to avoid division by zero, γ is the scale value that needs to be trained in the neural network, and β is the shift value that needs to be trained in the neural network. The pooling size of the Max-pooling and stride are (2, 2) and 2, respectively.

After the last Max-pooling layer, two FCNs are connected. The activation function of the first and second FCN is LeakyReLU and SoftMax. The formula of SoftMax is shown as follows:

(5) $$f{\left(x\right)}_{i}=\frac{e^{x_{i}}}{\sum_{k=1}^{K}e^{x_{k}}},\;for\;i=1,\ldots ,K$$

where x is the input vector of SoftMax, and K is the dimension of x. Through this formula, the input vector elements will be scaled between 0 and 1, and the summary of the elements is one.

There are three different types of input data, but the neural network structure is the same. The first CNN has a filter size of (5, 5) with 32 kernels; the second CNN has a filter size of (3, 3) with 64 kernels; the first FCN has 512 neuron size; the output layer has two neuron size. Due to the different sizes of the input data, the training number of the parameters is not the same. The difference in the training parameter of the three models is shown in Table 2. As shown in Table 2, according to the input size of the model, the number of parameters can be increased to four times the most minor size input. Below, we also have to consider whether adding that much computational cost is worth it. The discussion of computation power is in the next section.

The loss function of the models is categorical cross-entropy, as shown following:

(6) $$loss=-\overset{K}{\underset{i=1}{\sum }}y_{i}\cdot \log{\dot{y}}_{i}$$

where ${\dot{y}}_{i}$ is the *i*th scalar value in the model output, $y_{i}$ is the corresponding target value, and K is the number of scalar values in the model output. The optimizer is stochastic gradient descent [61,62]. There is a learning schedule for the optimizer to avoid gradient vanishing. In the fusion model, the initial learning rate, decay step, and decay rate of the learning schedule are 0.01, 1000, and 0.9, respectively. In the eye model, the initial learning rate, decay step, and decay rate of the learning schedule are 0.01, 1000, and 0.75, respectively. In the eye model, the initial learning rate, decay step, and decay rate of the learning schedule are 0.01, 1000, and 0.75, respectively. In addition, an early stop function is settled at 10,000 steps for the training process to avoid overfitting.

**Figure 4:**
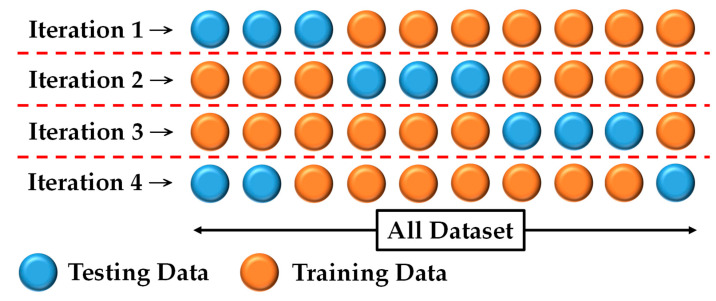
The K-fold cross-validation of this work.

K-fold cross-validation is settled in the training process, which is also settled in [50]. The total dataset of UTA-RLDD was split in a ratio of 7 to 3 as shown in Figure 4. Therefore, there were four iterations in the whole training process. The accuracy rates were shown by the average accuracy rates of the four iterations. The ROC curve and AUC were shown by the best AUC of the four iterations. Through the K-Fold cross-validation, it has been ensured that the model has not resulted in an overestimation of any accuracy index.

### 3.2. Gradient-Weighted Class Activation Mapping

The Grad-CAM is an improvement of class activation mapping (CAM) [63]. The CAM is an algorithm that explains how neural networks judge the input data. The output of the CAM is a heatmap highlighting the attention of neural networks. In the design of the CAM, there is a limitation that the last layer must be a global average pooling layer to replace the popular used fully connected layer. The formula of the CAM is shown as follows:

(7) $$S_{c}=\underset{k}{\sum }\omega_{k}^{c}\frac{1}{Z}\underset{i}{\sum }\underset{j}{\sum }A_{i,j}^{k}=\frac{1}{Z}\underset{i}{\sum }\underset{j}{\sum }\underset{k}{\sum }\omega_{k}^{c}A_{i,j}^{k}\;L_{CAM}^{c}=\underset{k}{\sum }\omega_{k}^{c}A_{i,j}^{k}$$

where A is the last CNN output, k is the channel number of the last CNN, c is the category, $\omega_{k}^{c}$ is the class feature weight of $A^{k}$, and Z is the value of multiplying the width and height of the final CNN output.

Although the CAM can be used to explain the judgment of neural networks, the limitation of adding a global average pooling layer often reduces the accuracy of the models. Therefore, the Grad-CAM is produced to improve the CAM. In the formula of the Grad-CAM, α is defined as follows:

(8) $$\alpha_{k}^{c}=\frac{1}{Z}\underset{i}{\sum }\underset{j}{\sum }\frac{\partial y^{c}}{\partial A_{i,j}^{k}}$$

where $y^{c}$ is the prediction score of the category c, and $\alpha_{k}^{c}$ is the specific weight for $A^{k}$. Therefore, the final formula of Grad-CAM is shown as follows:

(9) $$L_{Grad-CAM}^{C}=ReLU\left(\underset{k}{\sum }\alpha_{k}^{c}A^{k}\right)$$

Through formula (8), it is known that $\alpha_{k}^{c}$ is the value of backpropagating the prediction score $y^{c}$ on category c. Then, through the backpropagation, the importance of $A^{k}$ is calculated by the gradient. At last, the data of each channel of the feature layer A are weighted and summed through α, and finally, the Grad-CAM is obtained through the ReLU. The ReLU is used to filter out the negative pixels. The heatmap of Grad-CAM turns out after calculating formula (9). However, the heatmap is often smaller than the original input image. Therefore, the heatmap needs to be up-sampled to be able to draw on the original input image.

### 3.3. Feature Analysis Methods

This work presents two feature analysis methods, including feature visualization and KNN-Sigma. The feature visualization shows the output of the CNN layer and first FCN. The feature visualization has been implemented in our previous works [64,65]. The concept of feature visualization is to extract the last output of FCN and process the output by principal component analysis (PCA) [66]. The implementation of PCA is based on a covariance matrix shown as follows:

(10) $$C=\frac{1}{m}XX^{T}$$

where m is the dimension of the vector X. After obtaining the eigenvalues and eigenvector, the eigenvector should be sorted according to the eigenvalues by row. Finally, the output of PCA can be obtained as follows:

(11) Y=PX

where P is the sorted eigenvector.

Although most of the explanations of feature visualization are reasonable, sometimes feature visualization is still very complex and hard to explain [64,65]. Therefore, a method for evaluating feature distribution, KNN-Sigma, is proposed in this work. The concept of KNN-Sigma is based on the unsupervised computing clustering of K-nearest neighbors (KNN) [67] to quantify the homogeneous centrality and heterogeneous separation between features. The neighborhood of the feature set is shown as follows:

(12) $$T_{k}\left(x,U\right)=\left\{X\subseteq U\;|\;\left|X\right|=k,\forall x_{i}\in X\;and\;x_{j}\in U\;\backslash \;X,\delta \left(x,x_{i}\right)\leq \delta \left(x,x_{j}\right)\right\}$$

where U is the feature set $\left\{x_{1},x_{2},x_{3},\ldots ,x_{n}\right\}$, δ is the Euclidean distance, $\left|X\right|$ donates the cardinality of X. Therefore, the ε-neighborhood of x in U is given by:

(13) $$n_{\varepsilon }\left(x,U\right)=\left\{x_{i}\in U\;|\;\delta \left(x,x_{i}\right)\leq \varepsilon \right\}$$

The set of instances that are closer to x than $x_{i}$ is given by:

(14) $$c\left(x,x_{i},U\right)=\left\{x_{j}\in U\;|\;\delta \left(x,x_{j}\right)<\delta \left(x,x_{i}\right)\right\}$$

In order to deal with domain values that reach strictly or exceed *k* neighbors, the KNN threshold of x w.r.t U is given by:

(15) $$\varepsilon \left(k,x,U\right)=\underset{\left|n_{\varepsilon }\left(x,U\right)\right|\geq k}{\min}\varepsilon$$

Therefore, the extend k nearest neighbors of x in U is given by:

(16) $$\tau_{k}\left(x,U\right)=\left\{x_{i}\in U\;|\;\delta \left(x,x_{i}\right)\leq \varepsilon \left(k,x,U\right)\right\}$$

By voting according to the neighborhood, the class with the highest support rate can be obtained, which is the final classification shown as follow:

(17) $$R\left(x,\tau_{k}\left(x,U\right)\right)=\underset{c}{\mathrm{argmax}}\left|\left\{x_{i}\in \tau_{k}\left(x,U\right)\;|\;d\left(x_{i}\right)=c\right\}\right|$$

where $d\left(x_{i}\right)$ is the category of $x_{i}$. The concept of KNN-Sigma is to calculate the accuracy of all k and sum the final classification individually according to the percentage of k. The formula can be shown as follow:

(18) $$P_{I=100\left(\frac{k}{n}\right)+\frac{1}{2}}=\overset{n}{\underset{k}{\sum }}R_{k}$$

where P is a sum of the classification in aggregate of all *k*. The sum is used to observe the homogeneous centrality and heterogeneous separation of the extracted feature by plotting the curve of P.

## 4. Results and Discussion

### 4.1. Drowsiness Recognition

**Figure 5:**
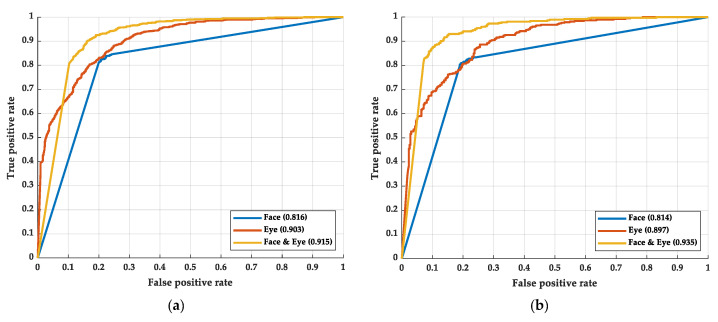
The ROC curve of the experimental results: (**a**) the results of the training process and (**b**) the results of the testing process.

The performance of three different methods is observed by some indexes, including the receiver operating characteristic curve (ROC curve) [68], the area under the ROC curve (AUC) [69], accuracy rate, and FPS. The ROC curve and AUC are shown in Figure 5. Figure 5a shows the ROC curve of the training process; Figure 5b shows the ROC curve of the testing process.

As shown in Figure 5, in the training process, the AUC of the face, eye, and fusion models are 0.816, 0.903, and 0.915, respectively. In the testing process, the AUC of the face, eye, and fusion models are 0.814, 0.897, and 0.935, respectively. The difference in AUCs between the training and testing process is not apparent, which means there is small overfitting in the models. Among the models, the face model has the smallest AUC. The AUC of the face model is even smaller than that of the eye model. We believe this may be caused by the face model mislearning the face recognition system and not learning the drowsiness recognition. The Grad-CAM is implemented to prove our hypothesis in the next stage. By simplifying the features of the face so that it leaves only the image of the eyes, the model learning face recognition can be avoided. After removing the information except for eye information from the face image, the AUC and ROC curve performed better than applying the whole face image.

At the same time, we found that the AUC and ROC curve can be further improved by fusing the eye and face images. In fusion, the proportion of eye images is more than that of face images. We speculate that the model will judge drowsiness based on eye features. However, if the model is further informed about the appearance of the face corresponding to this eye, judgment accuracy can be increased. After all, the eyes of different faces may have different features of drowsiness.

The accuracy rate and FPS are shown in Table 3. In the training process, the average accuracy rate of face, eye, and fusion models is 78.54%, 80.72%, and 86.84, respectively. In the testing process, the average accuracy rate of the face, eye, and fusion models are 77.66%, 79.74%, and 88.67%, respectively. The difference in average accuracy between the training and testing process is not apparent, which showed less overfitting within the three models. The accuracy of the eye model is greater than that of the face model, and the performance is reasonable according to the AUC and ROC curve. Although, while recognizing drowsy participants, the accuracy rate of the eye model is lower than that of the face model.

On the other hand, while recognizing alert participants, the accuracy rate of the eye model is greater than that of the face model. Therefore, the average accuracy of the eye model is still higher than that of the face model. Even the FPS performance of the eye model is greater than that of the face model. This comparison experiment shows that processing the specific feature can improve the performance of models.

**Figure 6:**
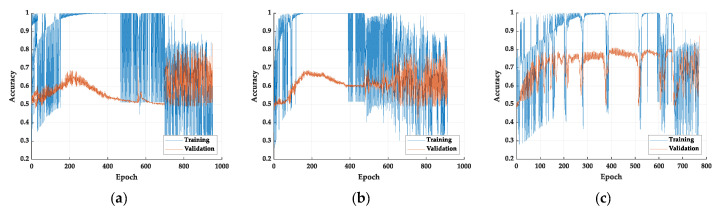
The accuracy vs. epochs of the training process: (**a**) face model, (**b**) eye model, and (**c**) fusion model.

Among the three models, the fusion model has the best performance in accuracy rate. However, the performance of FPS is the worst of the three models. The FPS of the face, eye, and fusion models are 18.7, 23.9, and 17.9, respectively. In the fusion model, the image of the face is not eliminated but is used as an additional item to increase the model accuracy. The eye features in the image are magnified, allowing the model to learn features of drowsiness rather than face recognition. We believe that the fusion of a small face to the model allows the model to determine further the drowsy eye features of various races of faces. We can observe the Grad-GAM of this model that helps realize whether the model has judged based on facial features. As for the accuracy vs. epoch, the training process is shown in Figure 6.

**Figure 7:**
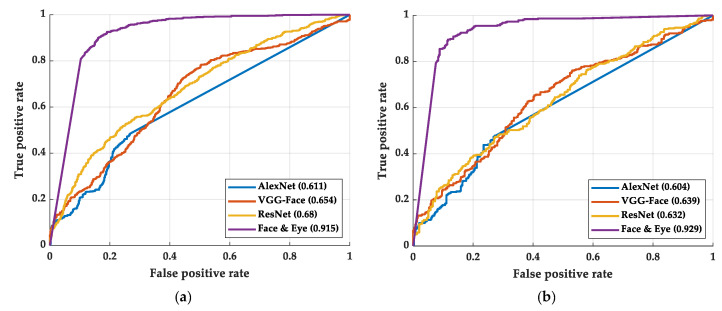
The AUC and ROC curve of the benchmark methods and our best method: (**a**) training process and (**b**) testing process.

Previously, most of the researchers presented models or methods only but did not go deep and analyze the neurons in the model. However, to compare our results with another method, we compare our accuracy rates with Alexnet [39], VGGFaceNet [46], and ResNet [41]. The ROC curves and AUCs are shown in Figure 7. As shown in Figure 7, our methods show a large advantage over the benchmark models. Table 4 shows the accuracy rate of the benchmark models and our best model. As shown in Table 4, our methods also show a large advantage over the benchmark models.

### 4.2. Gradient-Weighted Class Activation Mapping

**Figure 8:**
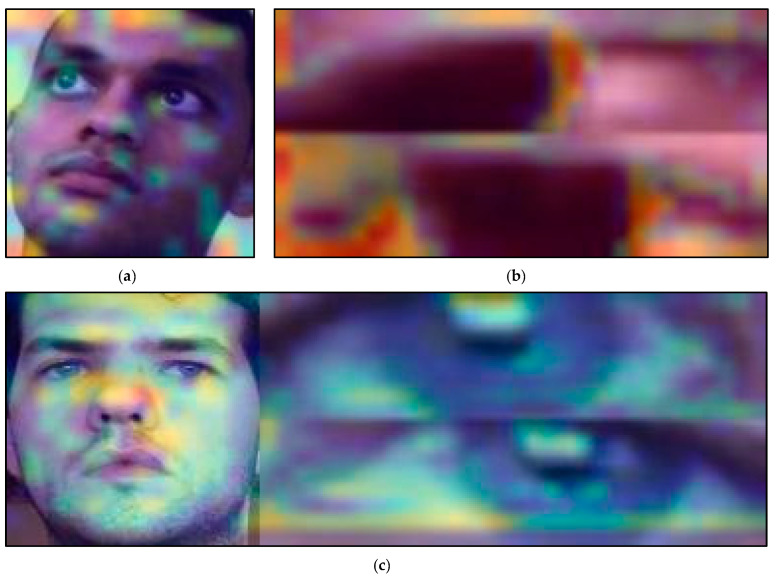
The Grad-CAMs of the three models: (**a**) face model, (**b**) eye model, and (**c**) fusion model. (The ID numbers of the participants are 23, 10, and 2 in the UTA-RLDD. The participants claim to allow their images to be published in research papers.)

In this part, the Grad-CAMs of this work are discussed. The Grad-CAMs of the three models are shown in Figure 8. Figure 8a shows the Grad-CAM of the face model; Figure 8b shows the Grad-CAM of the eye model; Figure 8c shows the Grad-CAM of the fusion model. The heatmap that covers the image shows the attention of the model. If the power of the heatmap is higher (red), the attention is more substantial. On the other hand, if the power of the heatmap is lower (blue), the attention is weaker.

As shown in Figure 8a, the model has not paid much attention to the eyes. The attention is very scattered, and it is similar to measuring the face mesh. It is generally believed that the most crucial features for drowsiness analysis should be the eye features. However, the model shows no attention to the eye features. We believe that using face alone for drowsiness recognition may cause the model to learn incorrectly, resulting in its learning as face recognition.

As shown in Figure 8b, by recognizing only the eye images, the recognition of face mesh can be avoided and make the model focus on the eye features. Unlike the results in Figure 8a, the model focus on the white of the eyes, which is reasonable. Drowsiness is often accompanied by red eye features [70]. The Grad-CAM of the eye model is more reasonable than the face model and makes the performance more significant than the face model in the final accuracy rate.

As shown in Figure 8c, the attention of the fusion model is distributed on the face and eye image. The attention of the face image is still similar to the face model. There is weak attention on the eyes in the face image. However, in the eye images, the model focuses on the white of the eyes and recognizes if there are any red eyes in the image. Using only eye features to identify drowsiness yields good results but adding facial features can further improve its accuracy. We speculate that the neural network further determines the drowsy eye feature corresponding to each face corresponding to the gender and race of the subjects. Different gender or race may have different features of ocular drowsiness.

### 4.3. Feature Analysis

**Figure 9:**
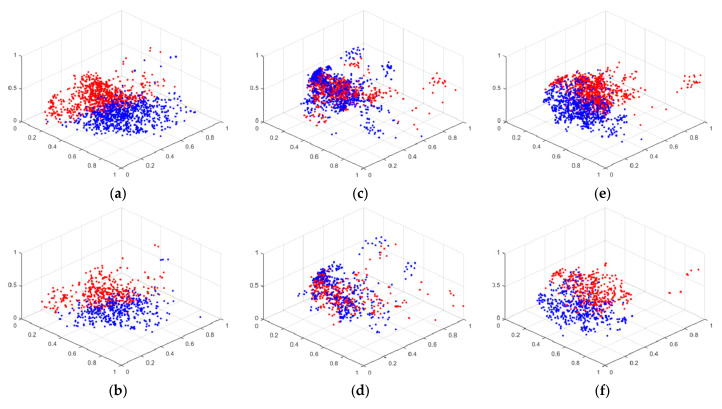
The feature visualization of the experiment with face and eye model: (**a**) training with face model, (**b**) testing with face model, (**c**) training with eye model, (**d**) testing with eye model, (**e**) training with fusion model, and (**f**) testing with fusion model.

In this part, the feature visualization and KNN-Sigma are discussed to compare the homogeneous concentration and heterogeneous separation of each model. In Figure 9, the feature visualizations are shown. The red spots are the features of drowsiness, and the blue spots are the features of alert. The features are the extraction of the last FCN processed by PCA. The component parameter of PCA is three. Figure 9a,b show the feature visualization of the face model of training and testing results, respectively. Figure 9c,d shows the feature visualization of the eye model of training and testing results, respectively. Figure 9e,f show the feature visualization of the fusion model of training and testing results, respectively. There is not much difference between the training and testing feature visualization, which also shows the same conclusion of the accuracy results with no overfitting.

As shown in Figure 9, the homogeneous concentration and heterogeneous separation of the feature points in the face and fusion models are similar. Both models clustered red and blue features well. Although the accuracy rate of the eye model performs better than that of the face model, the feature visualization of the eye model is not as good as that of the face model. Feature visualization can explain the feature extraction ability of the model, but it does not have sufficient quantification power. When observing the distribution of features, it is often necessary to follow the subjective judgment of the observer. Especially when the two feature visualization results are very similar, it is difficult for the observer to distinguish which feature visualization ability is the best. In addition, feature visualization does not explain why the distribution of eye features appears to be poor, but the recognition accuracy is high. Therefore, KNN-Sigma is proposed in this work to improve the explanation of feature extraction.

**Figure 10:**
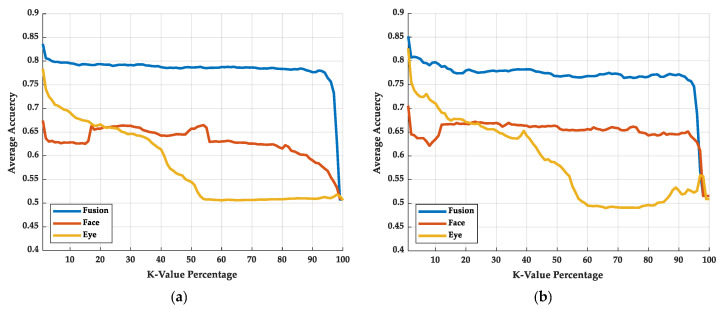
The KNN-Sigma results: (**a**) training results and (**b**) testing results.

In Figure 10, the results of KNN-Sigma are shown. Figure 10a,b shows the training and testing results of KNN-Sigma, respectively. With KNN-Sigma, we can try to quantify the pros and cons of feature distributions. According to the curve in Figure 10, when the K-value percentage is small, the average accuracy shows the homogeneous concentration of extracted features; when the K-value percentage is large, the average accuracy shows the heterogeneous separation of extracted features. There is not much difference between the training and testing curve of KNN-Sigma, which also shows the same conclusion of the accuracy results with no overfitting.

In Figure 10a,b, the curve of the fusion model remains the one with the highest average accuracy rate in both low and high K-value percentages, which means the fusion model has the best homogeneous concentration and heterogeneous separation performance. In the low K-value percentage, the performance of the eye model is better than that of the face model. In contrast, in the high K-value percentage, the performance of the face model is better than that of the eye model, which means that the homogeneous concentration of the eye model is better than that of the face model, but the heterogeneous separation of the face model is better than that of eye model. The classification result will relate to the highest average accuracy of the KNN-Sigma. While the model has the highest average accuracy in the KNN-Sigma, the accuracy rate of the model is also the highest.

### 4.4. Classification of the Gender with Eye and Facial Image

**Figure 11:**
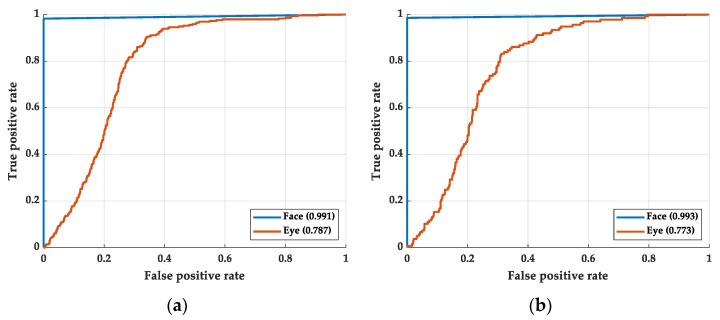
The ROC curve of the gender classification: (**a**) training process and (**b**) testing process.

In order to prove that gender features exist in the facial image but not in the eye image, we performed some additional experiments. We changed the outcome of the neural network from drowsiness to gender. The ROC curve is shown in Figure 11. Figure 11a shows the training process and Figure 11b shows the testing process. The deep learning structure of the gender classification is the same in this work. As shown in Figure 11, there is no obvious AUC or ROC curve difference between the training and testing process. The experimental shows that the relationship between eye and gender is less than that between face and gender. However, the relationship between eye and drowsiness is greater than that between face and drowsiness. Although eye feature is one of the features of facial features, distractions still occurred in the recognition of facial models.

**Figure 12:**
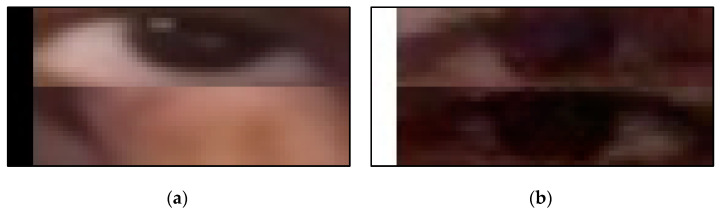
The remake fusion image with eye image and gender signal: (**a**) the eye image with male signal and (**b**) the eye image with the female gender.

**Figure 13:**
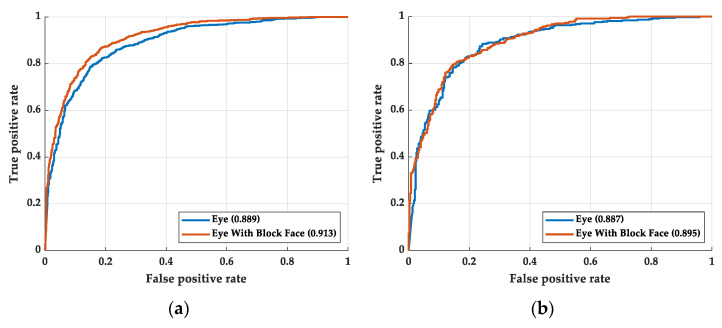
The ROC curve of the eye image with gender signal: (**a**) training process and (**b**) testing process.

In order to explain why the fusion model is attentive on the face without the eye, we made the face image into a binary image by gender classifying. The remake input image is shown in Figure 12. Figure 12a shows the eye image with a male signal and Figure 12b shows the eye image with a female signal. Figure 12 also shows how the image looks while the eye blinks. While the eye owner is male, the eye image is stacked with a zeros-value pixel image with a size of (5, 32). While the eye owner is female, the eye image is stacked with a 255-pixel value image with a size of (5, 32). This experiment shows that gender can help in recognizing drowsiness. The ROC curve of this experiment is shown in Figure 13. Figure 13a shows the training process and Figure 13b shows the testing process. As shown in Figure 13, the image with a gender signal has a better ROC curve and AUC than without a gender signal, no matter in the training process and testing process. However, the ROC curve and AUC of the image with gender signal are still not as good as concluding the whole face. There must be other unextracted features in the face image, such as race. Although proofing the race signal is useful for recognizing drowsiness is interesting, the UTA-RLDD does not provide the race of the participants. The proofing experiment required creating the new dataset and is considered to be our future work.

### 4.5. Hardware and Software

A workstation was used to complete the training process with a CPU of Intel (R) Core (TM) I7-5930K and four GPU of Nvidia GeForce TITAN X with scalable link interface (SLI) technology. The memory size of the training computer is 64 G with a type of DDR4. A different computer completes the testing process with an Intel (R) Core (TM) I7-9700K CPU and a GPU of Nvidia GeForce GTX 1080Ti. The memory size of the testing computer is 16 G with a type of DDR4.

Python mainly completed the software for this work. The deep learning package, TensorFlow, is used to implement the deep learning models of this work. The machine learning package, Scikit-learn, is used to implement the machine learning models of this work. Furthermore, all the figures in this work are drawn using MATLAB 2020a, Microsoft PowerPoint, and OpenCV because of the convenient drawing functions and neat image output.

## 5. Conclusions

In this work, we presented a comparative analysis of the face and eye features of drowsiness recognition. The comparison includes the accuracy rate and FPS, as well as the ROC curve, Grad-CAM, feature visualization, and KNN-Sigma of each model. In comparing the accuracy rate and ROC curve, we found that the eye model has a better performance than the face model. By comparing the Grad-CAM of both, we found that the attention of the face model has no relationship with drowsiness. We speculate that the face model did not learn drowsiness features but face recognition. Therefore, the muting of the face without the eyes reduces the distraction of the model and increases the accuracy rate of the model. The feature visualization result of the face model is better than that of the eye model.

Furthermore, by implementing KNN-Sigma, we found that the homogeneous concentration of the eye model is better than that of the face model. Among the three models, the fusion model with the mix of both signals shows the best accuracy rate and ROC curve. The accuracy rate of the fusion model increased by 11.01% and 8.93% from the face model and eye model, respectively; the AUC of the fusion model increased by 0.121 and 0.038 from the face and eye model, respectively. Among the models, the fusion model also shows the best KNN-Sigma. Regardless of the case with a low or high K-value percentage, the average accuracy of the fusion model is the highest. We believe that the methods demonstrated in this study allow deep learning models to understand driver drowsiness. If these methods can be implemented as an innovative function of drowsiness detection and alert in advanced driver-assistance systems, they can potentially improve traffic safety.

In the future, we aim to explore the enhancement of signals to compare the AI learning pattern. EEG is a more stable signal than an image signal. Although it is hard to measure the EEG of the driver with current technologies, the estimation of the EGG signals can potentially offer the ground truth of the drowsiness. The driving pattern is another considerable signal for drowsiness estimation. However, it is a very high-risk experiment to measure the driving patterns of drowsy participants in real-world driving. This experiment can only be conducted in a closed experimental setup or a driving simulator.

## Figures and Tables

**Table 1:** The Karolinska sleepiness scale levels and the classification of UTA-RLDD.

Levels	KKS Definitions	UTA-RLDD Definitions
Level 1	Extremely alert	Alert level
Level 2	Very alert	
Level 3	Alert	
Level 4	Rather alert	
Level 5	Neither alter nor sleepy	
Level 6	Some signs of sleepiness	Low vigilance level
Level 7	Sleepy, but no effort to keep alert	
Level 8	Sleepy, but some effort to keep alert	Drowsy level
Level 9	Very sleepy, significant effort to keep alert, fighting sleep	

**Table 2:** The number of parameters in the training model with different types of input.

Layers	Parameters in Neural Network		
Input layer	Face (28, 28, 3)	Eye (32, 64, 3)	Fusion (32, 96, 3)
BN	12	12	12
CNN (32 @ 5 × 5)	2432	2432	2432
BN	128	128	128
Max-Pooling	0	0	0
CNN (64 @ 3 × 3)	18,496	18,496	18,496
BN	256	256	256
Max-Pooling	0	0	0
FCN (512)	1,606,144	4,194,816	6,291,968
BN	2048	2048	2048
Output layer (2)	1026	1026	1026
Trainable Parameter	1,629,320	4,217,992	6,315,144
Total Parameter	1,630,542	4,219,214	6,316,366

**Table 3:** The accuracy rate and frame per second of the experiment with the operation point of 0.5.

	Status	Training Process			Testing Process			FPS
Method		Alert	Drowsy	Average	Alert	Drowsy	Average	
Face		75.88%	81.14%	78.54%	75.77%	79.63%	77.66%	18.7
Eye		84.82%	76.71%	80.72%	82.40%	76.98%	79.74%	23.9
Fusion		82.66%	90.90%	86.84%	84.85%	92.74%	88.67%	17.9

**Table 4:** The accuracy comparison of our methods and the benchmark models with the operation point of 0.5.

	Status	Training Process			Testing Process		
Method		Alert	Drowsy	Average	Alert	Drowsy	Average
AlexNet [39]		86.83%	23.26%	55.05%	85.86%	23.39%	54.63%
VGGFaceNet [46]		64.54%	57.14%	60.84%	62.88%	60.22%	61.55%
ResNet [41]		66.36%	57.02%	61.69%	63.38%	51.34%	57.36%
Our Fusion Net		82.66%	90.90%	86.84%	84.85%	92.74%	88.67%
